# Research Products Beyond the Research Paper: Reflections on User-Centered Evidence Synthesis From SeroTracker

**DOI:** 10.3389/frma.2022.881250

**Published:** 2022-05-19

**Authors:** Tingting Yan, Rahul K. Arora

**Affiliations:** ^1^Temerty Faculty of Medicine, University of Toronto, Toronto, ON, Canada; ^2^Center for Health Informatics, University of Calgary, Calgary, AB, Canada; ^3^Institute of Biomedical Engineering, University of Oxford, Oxford, United Kingdom

**Keywords:** evidence synthesis, dashboards, user-centered design, scientific communication, open data

## Introduction

Peer-reviewed scientific papers have become near-synonymous with credible scientific communication, both for primary research and for evidence syntheses (Goldbeck-Wood, 1999). While these papers benefit from peer review, they are also largely static outputs, and are not necessarily the best format for continuous updates, dynamic data exploration, or secondary data use.

COVID-19 has highlighted these challenges for evidence synthesis, as we struggle to keep up with an ever-evolving evidence base (Convenes, 2022). However, thousands of COVID-19 systematic reviews were registered–in some cases, more systematic reviews than primary studies (Pérez-Gaxiola et al., 2021). Of these, many reviews rapidly became outdated, and a small fraction had to be curated as the highest-quality efforts (McMaster Health Forum, 2021a).

At the same time, the pandemic has incubated a new generation of evidence synthesis products, including and beyond the research paper (McMaster Health Forum, 2021b; Convenes, 2022). We have seen more regularly-updated systematic reviews and rapid reviews designed to be responsive to decision-maker needs (Macdonald et al., 2020). Exceptional science journalism has helped synthesize scientific advances for the lay public (Yong, 2021). Finally, several dashboards have synthesized complex evidence across a variety of sources, such as the Oxford Covid-19 Government Response Tracker (Hale et al., 2021), the COVID-NMA systematic review dashboard of clinical trials (Boutron et al., 2020), and our project, SeroTracker (Arora et al., 2021). These evidence synthesis products are designed to meet user and decision-maker needs: curating, analyzing, and disseminating heterogeneous data in near-real-time.

In this piece, we will discuss our experiences building SeroTracker, a systematic review, dashboard, and data platform for SARS-CoV-2 seroprevalence studies. We propose a user-centered model for evidence synthesis, describe the challenges we faced in implementing this model, and reflect on questions for the scientific community to consider as these efforts become more commonplace.

## SeroTracker as an Example of User-Centered Evidence Synthesis

Early in the pandemic, many scientists started to publish seroprevalence studies–population-based antibody testing investigations that measure the prevalence of SARS-CoV-2 antibodies and help map the extent of SARS-CoV-2 infection and immunity (Bobrovitz et al., 2020). However, the results from these studies were published across research papers, preprints, and the news, with no unified data source.

Recognizing this gap, we built SeroTracker, a dashboard and data platform for SARS-CoV-2 serosurveys (Arora et al., 2021). SeroTracker rigorously curates and critically appraises SARS-CoV-2 seroprevalence data through an ongoing systematic review. To provide up-to-date evidence, we search for new published papers, preprints, and government reports weekly and update our dashboard continuously. We provide interactive analyses and open data on SeroTracker.com, aiming to enable rapid access and integration into other modeling and research efforts. The SeroTracker platform today includes data from over 3400 seroprevalence studies in 137 countries and territories, and has users across sectors including research groups, global public health organizations, the media, and the private sector (SeroTracker, 2022).

Reflecting on our work, we believe that SeroTracker's value has resulted from adopting a user-centered approach. We started by understanding users' needs through interviews and meetings, which led us to combine ideas from systematic reviews and data visualization to build a novel research product. We continuously engaged end-users throughout our design process, through both formal and informal quality improvement cycles. The user-centered design thinking approach has long been successfully applied in engineering (Razzouk and Shute, 2012), and is reflected by other widely-used health data projects such as Our World In Data and OpenPrescribing (Curtis and Goldacre, 2018; Mathieu et al., 2021).

## Key Considerations for User-Centered Evidence Synthesis

We faced numerous challenges while building SeroTracker, which raised key considerations for future user-centered evidence synthesis products. Here, we reflect on these considerations, our approaches to addressing them, and their implications for subsequent efforts.

### Considering Research Products Beyond the Research Paper May Help Drive More Value for Users

Scientific communication today centers around peer-reviewed research papers. These papers are a key form of scientific discourse, and peer review has an important role in helping validate research claims. However, journal publication can also be a slow process (Andersen et al., 2021; Donnici et al., 2022), hindering responses to a rapidly changing scientific landscape.

There is a broad spectrum of possible research products, and the peer-reviewed paper may not be the best product for every aim. A blog post might be more effective for lay communication, or a collaborative data repository to enable secondary analysis, and a pilot project to work toward implementing scientific findings.

To maximize the value of evidence synthesis for end users, we suggest taking a user-centered approach by aligning research products with end user needs. Such an approach directly optimizes for the real-world impact of research products. Researchers using this approach can learn from other fields where user-centered design is the norm. One example is engineering design, where engineers work with users to identify the problem, design a prototype, test it, and iterate on their solution (Meinel and Leifer, 2011; Razzouk and Shute, 2012).

In building SeroTracker, we worked closely with end users and decision-makers to design the platform, engaging them through user surveys, user interviews, and informal discussions. Users represented diverse organizations, and applied our data toward a variety of initiatives, from cataloging high-quality serosurveys aligned with the standardized UNITY protocol (World Health Organization, 2022), to modeling infection spread (Brazeau et al., 2020; COVID-19 Immunity Task Force, 2022); to estimating true pandemic death toll (Gamio and Glanz, 2021; The Economist, 2022). We used several strategies to reach users, including asking users to self-identify in future engagement when downloading our data, soliciting feedback from attendees at presentations we delivered, and direct outreach to individuals who publicly stated they were using our data. This approach ultimately led to numerous cycles of iteration, which improved all aspects of the tool, from details of our database schema to identifying highest-yield analyses.

Publication incentives may pose a challenge to adoption of this user-centered approach. Many researchers believe publication metrics play a role in hiring and promotion decisions (Abbott et al., 2010). These incentives can encourage researchers to prioritize producing publishable papers (Broad, 1981; Sarewitz, 2016), which indirectly conflicts with pursuing time-consuming and user-focused strategies. The broad applicability of user-focused products highlights the importance of incentivizing their development (Kucharski et al., 2020). Promisingly, the San Francisco Declaration on Research Assessment strongly advocates for alternative research products to be considered in evaluating researcher productivity (Cagan, 2013).

Prioritizing user needs means that we have published less often than we otherwise might have. However, in our view, this tradeoff has been worthwhile. For example, we learned from users that we should prioritize open and up-to-date data above all else. Providing this data has enabled many users to conduct their own novel analyses, magnifying the value of SeroTracker beyond what we could have achieved alone.

### Duplication of Efforts Slows Progress, Which Makes It Even More Crucial for Groups Answering Similar Research Questions to Collaborate

Traditional systematic reviews and meta-analyses may not be updated quickly enough during health crises. Inevitably, many initiatives scramble to answer similar research questions, with varying scopes and levels of rigor (Pérez-Gaxiola et al., 2021).

Contemporaneous with the creation of SeroTracker, several other initiatives pursued similar work–similar dashboards based at health agencies and other universities, summaries of seroprevalence research prepared by global infectious disease researchers, and crowdsourced Excel documents on Twitter with lists of seroprevalence articles.

Soon after, we recognized one another's efforts. In many cases, the seroprevalence initiatives generously contributed data to our open database, offered their kind support and instructive feedback, and in turn gained access to a unified database of evidence to drive their analyses. In this case, we happened to be best positioned to take our nascent effort forward. We expect to play a similar supporting role in many future evidence synthesis products.

Having several competing initiatives “race to publish first” implies duplicated efforts. It is the responsibility of all investigators to identify and communicate with groups answering similar research questions. Open data platforms can centralize the efforts of multiple groups, minimizing redundancy and enabling each group to carry out their work more efficiently (Kucharski et al., 2020).

### Researchers Should Aim to Document How They Integrate Data Systems, Visualization, and Decision-Makers

Each group building a user-centered evidence synthesis shares certain goals: regularly-updated data, ongoing synthesis of that data, and usefulness to decision-makers. However, the process of building these products is not well-documented. Until it is, investigators conducting their first such synthesis will face similar stumbling blocks.

Challenges we faced included managing large teams of human data extractors, creating a database system that is human-friendly but that can also feed a dashboard, and actively engaging end users in product development. As a “live” evidence synthesis effort, we also needed to quickly respond to major changes in the scientific landscape–for example, when vaccines became available, interpreting seroprevalence data became considerably more complex (Duarte et al., 2022).

When we started SeroTracker, it was difficult to find practical knowledge on how other data platforms were built or organized. Dashboard development is sometimes described in the medical literature, but these articles tend to focus on the novelty and technical architecture of the system being presented rather than key decision points in developing the tool (Wissel et al., 2020). We ultimately relied more on the management literature, which carefully documents this kind of implicit knowledge (Glaveski, 2021).

We are now working to document our own processes and learnings. We hope this documentation will help future developers of user-centered evidence syntheses and would also strongly encourage the developers of other dashboards to consider doing so.

## Discussion

We argue that design thinking should be used as a core tool for evidence synthesis. By engaging data users and decision-makers as “end users” of research products, we can thoughtfully apply the evidence synthesis process to create useful, targeted outputs, whether it be dashboards, living papers, or other scientific products. Ultimately, this approach will make evidence synthesis more effective in its end goal–to help governments, researchers, and institutions act quickly on new information.

It is essential to consider how to preserve the strengths of the existing scientific communication paradigm in a user-centered evidence synthesis approach. These well-established strengths include the rigor of pre-registration and reporting guidelines (e.g., PROSPERO, PRISMA), the validation and legitimacy provided by peer review, and clear dissemination mechanisms by which knowledge users can learn of new relevant work (Carpenter et al., 2014; Page et al., 2021).

Novel evidence synthesis products demand new standards for rigor and impact evaluation (Convenes, 2022). We posit several questions that we hope others can help explore. What does it mean to peer review a dashboard? What is the best way to measure the impact of these digital tools? Counting the number of page views is (perhaps too) simple, but it is much more challenging to measure impact through informing policy. How can we appropriately fund this sort of research infrastructure, which often does not directly fit the mandate of traditional academic funding sources (Hershberg, 2022)? And how do we make new products easily discoverable, to help researchers publicize their work and knowledge users find useful tools?

Through SeroTracker and other initiatives, we have seen the benefits of groups collaborating on widely accepted, open-access core datasets. Many different users can apply these datasets to their own research questions, reducing duplication of effort and enabling scientists to focus on new analyses. Closed datasets privilege those with access to said data. In contrast, the open nature of this data creates opportunities for any researcher to participate–particularly those without traditional forms of power.

We expect user-centered evidence synthesis to become even more relevant as the volume of data grows, researchers become more technologically adept, and health emergencies demand us to close the time gap between data and decisions (Convenes, 2022). In the wake of COVID-19, we have the opportunity to move toward user-centered evidence syntheses purpose-built for maximum impact.

## Author Contributions

TY and RA jointly conceptualized, wrote, and revised this piece. Both authors contributed to the article and approved the submitted version.

## Funding

Separate from this work, SeroTracker receives funding from the Public Health Agency of Canada through Canada's COVID-19 Immunity Task Force, the World Health Organization Health Emergencies Programme, the Robert Koch Institute, and the Canadian Medical Association Joule Innovation Fund. These funding sources had no role in this study, including conceptualization, writing, or the decision to submit for publication. This manuscript does not necessarily reflect the views of the World Health Organization or any other funder.

## Conflict of Interest

RA was previously a Technical Consultant for the Bill and Melinda Gates Foundation Strategic Investment Fund, is a minority shareholder of Alethea Medical, and was a former Senior Policy Advisor at Health Canada. TY was a former Senior Policy Advisor at Health Canada. Each of these relationships is unrelated to the present work.

## Publisher's Note

All claims expressed in this article are solely those of the authors and do not necessarily represent those of their affiliated organizations, or those of the publisher, the editors and the reviewers. Any product that may be evaluated in this article, or claim that may be made by its manufacturer, is not guaranteed or endorsed by the publisher.
